# Performances of preoperative CT scan to predict the pTN stage for MSI/dMMR localized colon cancers

**DOI:** 10.1016/j.esmoop.2024.103678

**Published:** 2024-08-14

**Authors:** M. Duval, Q. Vanderbecq, V. Phou, B. Cervantes, L. Mas, J.-B. Bachet, C. Goumard, Y. Parc, T. André, J.H. Lefèvre, O. Lucidarme, L. Arrivé, R. Cohen, M. Wagner

**Affiliations:** 1 Sorbonne Université, Service d’Oncologie Médicale, Hôpital Saint Antoine, AP-HP, INSERM UMRS 938, Centre de Recherche Saint-Antoine, Equipe Instabilité des Microsatellites et Cancer, Equipe labellisée par la Ligue Nationale contre le Cancer, SIRIC CURAMUS, Paris; 2 Sorbonne Université, Service de Radiologie, Hôpital Saint Antoine, APHP, Laboratoire d’Imagerie Biomédicale, UMR 7371, UMR S 1146, Paris; 3 Sorbonne Université, Service d’Imagerie (SISU), Hôpital Pitié Salpêtrière, APHP, Laboratoire d’Imagerie Biomédicale, UMR 7371, UMR_S 1146, Paris; 4 Sorbonne Université, Service d’Hépato-gastroentérologie et Oncologie Digestive, Hôpital Pitié Salpêtrière, APHP, Paris; 5 Sorbonne Université, Service de Chirurgie Digestive, Hépatobiliaire et Transplantation Hépatique, Hôpital Pitié Salpêtrière, Centre de Recherche Saint-Antoine, INSERM UMR S-938, Paris; 6 Sorbonne Université, Service de Chirurgie Générale et Digestive, Hôpital Saint-Antoine, AP-HP, INSERM UMRS 938, Centre de Recherche Saint-Antoine, Equipe Instabilité des Microsatellites et Cancer, Equipe labellisée par la Ligue Nationale contre le Cancer, SIRIC CURAMUS, Paris, France

**Keywords:** colon cancer, MSI/dMMR, immune checkpoint inhibitor, neoadjuvant therapy, preoperative staging

## Abstract

**Background:**

Neoadjuvant immunotherapy emerges as a promising strategy for patients with localized colon cancer (CC) harboring microsatellite instability/mismatch repair deficiency (MSI/dMMR). The aim of this study is to evaluate the concordance between clinical cTN stage assessed by preoperative computed tomography (CT) scan and pTN stage of MSI/dMMR CC.

**Patients and methods:**

Consecutive patients diagnosed for localized MSI/dMMR CC and treated with upfront surgery between 2013 and 2022 in two French centers were eligible. Two independent radiologists, blinded to pathological findings, reviewed all preoperative CT scans and assessed cTN stage, with a third radiologist reviewing discordant cases. Radiological predictive diagnostic accuracy for pT4 and pN+ (N+ = N1 or N2) were calculated.

**Results:**

One hundred and thirteen patients were included (right CCs = 79%). CT scan diagnostic performances for pT4 were sensitivity (Se) = 33.3%; specificity (Sp) = 94.0%; positive predictive value (PPV) = 66.7%; and negative predictive value (NPV) = 79.6% and for pN+ were Se = 70.3%; Sp = 59.2%; PPV = 45.6%; and NPV = 80.4%. When pT-pN were combined, 37.5% of tumors identified as cT4 and/or cN+ were actually pT1-3 and pN0, and 23.1% of the pT4 and pN+ population was not identified as such radiologically.

**Conclusion:**

The ability of preoperative CT scan to predict pT and pN stages is limited for localized MSI/dMMR CCs. Reassessing neoadjuvant strategies’ benefit–risk balance in this population is needed.

## Highlights

• A large cohort of MSI/dMMR localized CCs (113) and CT scan reviewing by three expert blinded independent radiologists.
• On preoperative CT scan, 33% of cT4 tumors were actually pT1-3 and 66% of the pT4 population were not identified as such.
• On preoperative CT scan, 54% of cN-positive tumors were pN-negative and 30% of the pN-positive population were cN-negative.
• Preoperative CT scan has limited performances to correctly identify TN stages for MSI/dMMR CC.
• Other diagnostic/prognostic tools are needed to properly develop neoadjuvant strategies with immune checkpoint inhibitors.

## Introduction

Microsatellite instability/deficient mismatch repair (MSI/dMMR) tumors show a high level of lymphocyte infiltration, indicating an effective host immunologic response. This phenotype has emerged as a major predictive biomarker for the efficacy of immune checkpoint inhibitors (ICIs) in colon cancer (CC). It occurs in ∼15%-18% of stage II CC and 9%-10% of stage III CC and is associated with a favorable prognosis for stage II and stage III pN1 CC but with prognosis equivalent to that of microsatellite stable/MMR proficient (MSS/pMMR) stage III pN2 CC.^1^^,^^2^

Recently, neoadjuvant immunotherapy has emerged as a promising strategy for the management of patients in the localized MSI/dMMR CC setting.^3^, ^4^, ^5^, ^6^

Assessing the benefit–risk balance of these strategies becomes a key issue, even more since the disease-associated prognosis mainly relies on pTN classification that is unknown in the neoadjuvant setting. Data are scarce regarding the accuracy of preoperative computed tomography (CT) to predict pT4 or pN+ stages for the MSI/dMMR CC. Several studies have evaluated the performances of preoperative CT scan to predict pTN stage, but without data regarding MMR status.^7^^,^^8^ Yet, MSI/dMMR tumors are characterized by high immune infiltrate, with notably an increase in the number of analyzed lymph nodes in surgical specimens. Therefore, they might be at higher risk of overstaging by preoperative CT scan.^9^^,^^10^ This is reinforced by a recent study showing that right-sided MSI/dMMR CCs are associated with larger lymph nodes.^11^

Given the above, we aimed at evaluating the diagnostic performances of preoperative CT scan to predict pTN stage specifically in patients with localized MSI/dMMR CC.

## Patients and methods

### Cohort selection and study variables

All consecutive localized MSI/dMMR patients who underwent primary tumor resection between 1 January 2013 and 31 December 2022 at two French university hospital centers were identified. MSI/dMMR status was confirmed by immunohistochemistry (IHC) and/or PCR. Inclusion criteria were (i) patients who underwent colectomy due to stage I to III MSI/dMMR CC and (ii) preoperative thoraco-abdomino-pelvic (TAP) CT scan with contrast injection (IV+) carried out within 60 days before surgery. Exclusion criteria were (i) neoadjuvant treatment or systemic treatment for other malignancies <5 years; (ii) incomplete/unknown pathological staging; (iii) multiple synchronous CCs; (iv) perforating tumor; (v) only non-contrast CT acquisition and/or non-available contrast-enhanced TAP CT scan at baseline; and (vi) tumor located in the middle to distal portion of the rectum.

### Preoperative CT scan assessment

Two radiology fellows specialized in digestive imaging, blinded to the definitive pTN stage, independently reviewed all preoperative baseline CT scan for cT stage and cN stage using a predefined methodology. T stage was evaluated as follows: T1-2—mass in colon lumen limited to the bowel wall with clear pericolic fat on CT; T3—tumor that demonstrated smooth or nodular extension beyond the normal contour of the bowel wall; T4—tumor that extends into adjacent peritoneum or grows into other adjacent tissues or organ. Lymph nodes were considered pathological (cN+) if presented with the following: internal heterogeneity and/or irregular borders regardless of the size, a size ≥10 mm, aggregation with healthy morphological appearance and size ≥5 mm and <10 mm or a combination of the above criteria.^7^^,^^11^^,^^12^ A third independent senior expert radiologist, who was blinded to the other reader’s classification, reviewed the discordant cases.

### Statistical analysis

Endpoints were sensitivity, specificity, and positive and negative predictive value of CT scan to predict pT stage (pT1-2-T3; pT4) and pN stage (pN0; pN+). Cohen’s kappa (κ) on T and N stages was calculated between the two radiology fellows.

R statistical Software v.4.2.0 was used to carry out all analyses.

## Results

### Population

From 291 identified MSI/dMMR CC patients, 178 patients were excluded for the following reasons: CT scan unavailable for missing medical record (*n* = 114), CT assessment not meeting pre-specified criteria (*n* = 23), cancer located at middle to distal portion of rectum (*n* = 16), multiple synchronous CC (*n* = 15), history of other malignancy <5 years and/or previous treatment (*n* = 7), and perforation (*n* = 3) (see flow chart in Figure S1 in the Supplementary Material, available at https://doi.org/10.1016/j.esmoop.2021.100138). The main clinical and disease characteristics are displayed in Table 1.

**Table 1:** Baseline characteristics of patients

Characteristics	Number of patients (%)
Study population	113 (100)
Sex	
Male	47 (41.6)
Female	66 (58.4)
Median age, years (range)	72 (25-94)
Median delay in days between CT and surgery (range)	18 (0-60)
Pathological tumor location	
Right colon	87 (77.0)
Transverse colon	9 (8.0)
Left colon	17 (15.0)
Pathological T classification	
pT1	2 (1.8)
pT2	16 (14.2)
pT3	65 (57.5)
pT4	30 (26.5)
Pathological N classification	
pN0	76 (67.3)
pN1	26 (23.0)
pN2	11 (9.7)
Pathological TN classification	
pT1-3 and pN0	61 (54.0)
pT4 and/or pN1-2	52 (46.0)

CT, computed tomography; N, node; T, tumor.

### Agreement between clinical and pathological T and N stages

**Figure 1:**
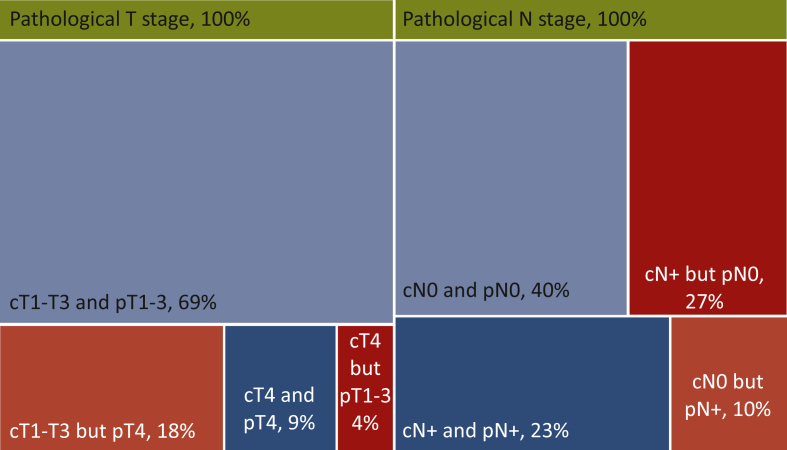
**Treemap of preoperative CT scan accuracy for pathological T stage and N stage.** Blue: correct evaluation by CT scan. Red: incorrect evaluation by CT scan. CT, computed tomography; N, node; T, tumor.

Tumors were radiologically assessed as cT1-2, cT3 and as cT4 in 32 (28.3%), 66 (58.4%) and 15 (13.3%) patients, respectively. Cohen’s κ was 0.62 for cT3-T4 versus cT1-T2/unseen tumors, 0.24 for cT4 versus cT1-T3/unseen tumors and 0.65 for cT4 and/or cN+ versus cT1-3N0/unseen tumors. Among the 15 tumors identified as cT4, 5 (33.3%) were pathologically staged as pT1-3. Twenty of the 30 pT4 lesions (66.6%) were not identified as such by preoperative CT scan (Figure 1 and Table 2). Exploratory data for pT4b tumors (*n* = 16) are described in Table S1 in the Supplementary Material, available at https://doi.org/10.1016/j.esmoop.2021.100138.

**Table 2:** Agreement between clinical and pathological T and N stages

Clinical stage	Pathological stage				Overestimation by CT scan	Correct	Underestimation by CT scan
T stage	pT1/2	pT3	pT4	Total	cT > pT	cT = pT	cT < pT
cT1/2	14	16	2	32	NA	43.8%	56.3%
cT3	4	44	18	66	6.1%	66.7%	27.3%
cT4	0	5	10	15	33.3%	66.7%	NA
Total	18	65	30	113			
N stage	pN0	pN+		Total	cN > pN	cN = pN	cN < pN
cN0	45	11		56	NA	80.4%	19.6%
cN+	31	26		57	54.4%	45.6%	NA
Total	76	37		113			
TN stage	pT1-3 and pN0	pT4 and/or pN+		Total	cTN > pTN	cTN = pTN	cTN < pTN
cT1-3 and cN0	37	12		49	NA	75.5%	24.4%
cT4 and/or cN0	24	40		64	37.5%	62.5%	NA
Total	61	52		113			

CT, computed tomography; N, node; T, tumor.

Tumors were radiologically assessed as cN0 and as cN+ in 56 (49.6%) and 57 (50.4%) patients, respectively. Cohen’s κ on N stage was 0.63. Among the 57 tumors identified as cN+, 31 (54.4%) were pathologically staged as pN0. Eleven of the 37 pN+ lesions (29.7%) were not identified as such by preoperative CT scan (Figure 1 and Table 2).

When T stage and N stage were combined, 37.5% of tumors identified as cT4 and/or cN+ were pathologically staged as pT1-3pN0 (24 out of 64), while 23.1% of the pT4 and/or pN+ population was not identified as such by preoperative CT scan (12 out of 52); (Figure 1 and Table 2).

Diagnostic performances of CT scan for pT, pN and pTN stages are summarized in Table 3.

**Table 3:** Diagnostic accuracy of CT scan

Performances	pT4	pN+	pT4 and/or N+	
Sensitivity (%)	33.3	70.3	76.9^a^	a95% CI (68.1-85.7)
Specificity (%)	94.0	59.2	60.7^a^	a95% CI (53.1-68.3)
Positive predictive value (%)	66.7	45.6	62.5^a^	a95% CI (53.9-71.1)
Negative predictive value (%)	79.6	80.4	75.5^a^	a95% CI (67.6-83.4)

CI, confidence interval; CT, computed tomography; N, node; T, tumor.

a Indicates the value to which the confidence interval refers.

Exploratory data by tumor location (right, transverse and left colon) are available in Tables S2-S4 in the Supplementary Material, available at https://doi.org/10.1016/j.esmoop.2021.100138.

## Discussion

This large bicentric cohort demonstrates the limited accuracy of preoperative CT scan for predicting the pathological pTN stage of MSI/dMMR CC. One-third of tumors classified as cT4 were in fact pT1-3; 54.4% of tumors identified as clinically stage III (i.e. cN+) were in fact stage II diseases (i.e. pN−). On the contrary, two-thirds of the pT4 population and almost one-third of the pathological stage III population were not identified as such by preoperative CT scan.

Focusing on the population usually targeted by clinical trials evaluating neoadjuvant immunotherapy in CC (i.e. T4N0 or stage III MSI/dMMR), our data showed 37.5% of the tumors clinically staged as cT4 and/or stage III are pathologically classified as pT1-3 and stage II (pN−), while almost one-fourth (12/52) of the pT4 and/or pN+ are not identified by preoperative CT scan (clinically staged as cT1-3 and cN0). Therefore, using clinical T and N stage as assessed by CT scan as an eligibility criteria for neoadjuvant immunotherapy may (i) miss a significant part of the population considered at risk of relapse and targeted by clinical trials, and (ii) induce overtreatment of MSI/dMMR CC patients. These results should be put into perspective with the better prognosis of MSI/dMMR over MSS/pMMR CC patients after tumor stage II surgical resection, non-negligible toxicity of ICIs and economic issues to determine the most appropriate benefit–risk balance.^2^^,^^13^ As they may address some of the concerns previously raised regarding risk stratification, future results from the registrational phase III study currently recruiting (NCT05855200—perioperative ICI in cT4N0/stage III CC randomized against standard care) will be looked at with great interest.

Besides, these eligibility radiological criteria are all the more questionable as the reproducibility of radiological assessment remains limited with Cohen’s κ < 0.70 in most favorable cases. In this regard, our data are in line with those from the FOxTROT study, with an interreader agreement of κ = 0.68 for classifying high-risk (T stage) tumors, and κ = 0.44 for differentiating N0 from N+ tumors.^14^

Although retrospective, our study, dedicated to MSI/dMMR CCs, shows multiple strengths, particularly the sample size, and the bicentric design with dual centralized blinded radiologic review with predefined criteria for cT and cN assessment.^7^^,^^11^^,^^12^ It is noteworthy that, these criteria were chosen to maximize the specificity to detect positive lymph node and avoid overtreatment for this population with overall better prognosis. This is even more important since MSI/dMMR tumors are known to be associated with immune reaction and a higher number of regional lymph nodes than for MSS/pMMR CC, especially in cases without lymph node metastasis.

Data about correlation between cTN and pTN stages using preoperative CT scan mostly concern unspecified MMR-status CCs. In a large systematic review and meta-analysis of 13-16 studies evaluating the diagnostic accuracy of CT scan in this unspecified setting, sensitivity and specificity were 77% and 70%, respectively, for detection of T3cd-T4 tumors and, 71% and 67%, respectively, for nodal involvement N+.^15^ However, the immunogenicity of MSI/dMMR tumors and the personalized therapeutic strategies for localized CCs require specific data with regard to MSI/dMMR status. For instance, in a small cohort of 44 localized dMMR CCs and 57 pMMR CCs, dMMR CCs were at higher risk of N overstaging.^9^

To summarize, our data show good sensitivity and specificity of CT to detect pT3-4 tumors but not pT4 alone (which may be more clinically relevant), and poor accuracy in detecting lymph node metastases, with overall diagnostic performances slightly worse than for MSS/pMMR CCs. New diagnostic tools, potentially based on radiomics, are needed to better predict pTN stage and tumor-associated prognosis. Research efforts on radiomics might provide significant improvements in the near future.^16^

## Conclusion

Preoperative CT scan shows limited reliability in predicting pT and pN stages, especially pT4 and pN+, for localized MSI/dMMR CCs. These results should be considered when assessing the benefit–risk balance of neoadjuvant immunotherapy strategies in this population.
