# Supplementary material for: Performances of preoperative CT scan to predict the pTN stage for MSI/dMMR localized colon cancers
Source: ESMO Open. 2024 Aug 14;9(8):103678. doi: 10.1016/j.esmoop.2024.103678 (PMC11374966; doi:10.1016/j.esmoop.2024.103678)
Supplement: Supplementary data [file mmc1.docx]

**SUPPLEMENTAL APPENDIX**

**Figure 1’.** Flow chart

**Inclusion criteria (n=291)**

- Colectomy due to non metastatic colon cancer regardless localization and pathology (jan 2013 – dec 2022)
- Information of MSI/dMMR status available

**Exclusion criteria (n=178)**

- Missing CT images or medical files (n=114)
- History of other malignancy < 5y and/or previous treatment (n=7)
- Cancer located at middle to distal portion of rectum (n= 16)
- CT assessment not meeting pre-specified criteria (n= 23)
- Initial perforation (n= 3)
- Multiple synchrone colon cancer (n= 15)

**FFinal study population (n=113)**

**Table 1’.** Agreement between clinical and pathological T stages over pT4b population

| **Pathological stage** | **Clinical stage** | | | | **Overestimation by CT scan** | **Correct** | **Underestimation by CT scan** |
| --- | --- | --- | --- | --- | --- | --- | --- |
| **T-stage** | cT1/2 | cT3 | cT4 | Total | cT>pT | cT=pT | cT<pT |
| pT4b | 0 | 7 | 9 | 16 | NA | 56.2 % | 43.7 % |

**Table 2’.** Agreement between clinical and pathological T and N stages over right colon cancers

| **Clinical stage** | **Pathological stage** | | | | **Overestimation by CT scan** | **Correct** | **Underestimation by CT scan** |
| --- | --- | --- | --- | --- | --- | --- | --- |
| **T-stage** | pT1/2 | pT3 | pT4 | Total | cT>pT | cT=pT | cT<pT |
| cT1/2 | 11 | 11 | 1 | 23 | NA | 47.8 % | 52.2 % |
| cT3 | 3 | 38 | 13 | 54 | 5.6 % | 70.4 % | 24.1 % |
| cT4 | 0 | 4 | 6 | 10 | 40.0 % | 60.0 % | NA |
| Total | 14 | 53 | 20 | 87 |  |  |  |
| **N-stage** | pN0 | pN+ | | Total | cN>pN | cN=pN | cN<pN |
| cN0 | 30 | 9 | | 39 | NA | 76.9 % | 23.1 % |
| cN+ | 28 | 20 | | 48 | 58.3 % | 41.7 % | NA |
| Total | 58 | 29 | | 87 |  |  |  |

**Table 3’.** Agreement between clinical and pathological T and N stages over left colon cancers

| **Clinical stage** | **Pathological stage** | | | | **Overestimation by CT scan** | **Correct** | **Underestimation by CT scan** |
| --- | --- | --- | --- | --- | --- | --- | --- |
| **T-stage** | pT1/2 | pT3 | pT4 | Total | cT>pT | cT=pT | cT<pT |
| cT1/2 | 3 | 4 | 1 | 8 | NA | 37.5 % | 62.5 % |
| cT3 | 0 | 3 | 3 | 6 | 0 % | 50 % | 50 % |
| cT4 | 0 | 0 | 3 | 3 | 0 % | 100 % | NA |
| Total | 3 | 7 | 7 | 17 |  |  |  |
| **N-stage** | pN0 | pN+ | | Total | cN>pN | cN=pN | cN<pN |
| cN0 | 11 | 1 | | 12 | NA | 91.7 % | 8.3 % |
| cN+ | 2 | 3 | | 5 | 40 % | 60 % | NA |
| Total | 13 | 4 | | 17 |  |  |  |

**Table 4’.** Agreement between clinical and pathological T and N stages over transverse cancers

| **Clinical stage** | **Pathological stage** | | | | **Overestimation by CT scan** | **Correct** | **Underestimation by CT scan** |
| --- | --- | --- | --- | --- | --- | --- | --- |
| **T-stage** | pT1/2 | pT3 | pT4 | Total | cT>pT | cT=pT | cT<pT |
| cT1/2 | 0 | 1 | 0 | 1 | NA | 0 % | 100 % |
| cT3 | 1 | 3 | 1 | 5 | 20 % | 60 % | 20 % |
| cT4 | 0 | 1 | 2 | 3 | 33.3 % | 66.6 % | NA |
| Total | 1 | 5 | 3 | 9 |  |  |  |
| **N-stage** | pN0 | pN+ | | Total | cN>pN | cN=pN | cN<pN |
| cN0 | 4 | 1 | | 5 | NA | 80 % | 20 % |
| cN+ | 1 | 3 | | 4 | 25 % | 75 % | NA |
| Total | 5 | 4 | | 9 |  |  |  |
